# Impact of sleeve gastrectomy on the periodontal status of patients with and without type 2 diabetes: a 1-year prospective real-world study

**DOI:** 10.3389/fendo.2024.1431728

**Published:** 2024-08-15

**Authors:** Xiaocheng Bi, Peikai Zhao, Teng Liu, Tao Zhu, Yuxuan Li, Sisi Xiong, Shaozhuang Liu, Xiaole Hu, Xin Huang

**Affiliations:** ^1^ Department of Periodontology, School and Hospital of Stomatology, Cheeloo College of Medicine, Shandong University & Shandong Key Laboratory of Oral Tissue Regeneration and Shandong Engineering Research Center of Dental Materials and Oral Tissue Regeneration and Shandong Provincial Clinical Research Center for Oral Diseases, Jinan, Shandong, China; ^2^ Division of Bariatric and Metabolic Surgery, Department of General Surgery, Qilu Hospital of Shandong University, Jinan, Shandong, China; ^3^ The First Clinical College, Shandong University, Jinan, Shandong, China; ^4^ State Key University Laboratory of Diabetes and Obesity Surgery, Shandong University, Jinan, Shandong, China; ^5^ Department of Operating Room, Qilu Hospital of Shandong University, Jinan, Shandong, China

**Keywords:** type 2 diabetes, periodontitis, obesity, sleeve gastrectomy, prospective studies

## Abstract

**Background:**

Periodontitis is a chronic inflammatory disease potentially associated with obesity and type 2 diabetes (T2D). Sleeve gastrectomy (SG) has shown substantial effect on weight loss and treatment of T2D. However, there is no direct evidence comparing the impact of SG on the periodontal status of patients with and without T2D.

**Objectives:**

To determine the impact of SG on the periodontal status of patients with and without T2D in a real-world setting.

**Methods:**

In a prospective and two-armed cohort design, participants who were scheduled for SG at an affiliated hospital between April 2022 and December 2022 were approached for eligibility. After a clinical evaluation and oral examination, those with periodontitis were included and further divided into the DM group (diabetic) and the Control group (non-diabetic) with a 1-year follow-up after surgery. The primary outcome was the periodontal status of patients at 12 months after SG. The secondary outcomes included weight loss, diabetes remission, and alterations in inflammatory markers for up to 1 year after SG.

**Results:**

Fifty-seven and 49 patients were included in the DM and the Control group, respectively. Before surgery, patients in the DM group had further worsened periodontal condition compared with those in the Control group. Accompanied by weight loss and glucose reduction, patients in both groups demonstrated significant decreases in plaque index (PLI) and bleeding index (BI) with no alterations in probing depth or clinical attachment loss for up to 1 year after SG. Even patients in the DM group achieved less TWL% (32.79 ± 6.20% vs. 37.95 ± 8.34, *P*<0.01), their periodontal condition had more substantial improvement with no significant difference in PLI and BI between groups at 1 year after SG. We also observed a significant reduction in the levels of high sensitive C-reactive protein and interleukin-6 in both groups at 1 year after SG.

**Conclusion:**

Both patients with and without T2D demonstrated improved periodontal status for up to 1 year after SG. Patients with T2D achieved less weight loss but a more substantial improvement in periodontal condition. The significant reduction in inflammatory biomarkers contributed to the improvement of periodontal status after SG.

## 1 Introduction

The prevalence of obesity and type 2 diabetes (T2D) continues to increase significantly in developed and developing countries. According to the latest survey, 42% of adults are with overweight or obesity globally (1), and approximately 537 million adults are affected by T2D worldwide (2).

Periodontitis is a non-communicable disease characterized by pathological loss of the periodontal ligament and alveolar bone (3). Severe periodontitis affects 11.2% of the world’s population, being the 6th most prevalent disease worldwide and the primary cause of tooth loss (4). Treatment of periodontitis aims to prevent further disease progression, reduce the risk of tooth loss, possibly restore lost periodontal tissue, and maintain a healthy periodontium (5).

Patients with obesity are considered a risk group for periodontitis. However, the importance of oral health in patients with obesity, especially those with morbid obesity, is commonly overlooked due to their other life-threatening comorbidities. The limited available evidence has demonstrated the periodontitis prevalence in patients with obesity and eligible for bariatric/metabolic surgery (BMS) ranges between 45% and 70% (6–8). In addition, there is an evident bidirectional relationship between T2D and periodontitis. Patients with T2D are reported to have significantly worse periodontal status and a 34% greater risk of developing periodontitis (9). Likewise, severe periodontitis increases the incidence of T2D by 53% (9). However, when we focus on the patients with T2D and eligible for BMS, there has been no direct evidence on their periodontal health status.

BMS has shown substantial effect on weight loss and the treatment of T2D (10, 11). Sleeve gastrectomy (SG) is the most commonly performed bariatric/metabolic procedure, accounting for approximately 62.5% of all primary BMS worldwide (12). Changes induced by surgery, including weight loss, a decrease in blood glucose, a reduction in systemic inflammation, alterations in eating habits and dietary choices, and remodeling of the oral microbiota, could have a significant impact on the periodontal status of patients. However, there has been no consensus in terms of the impact of BMS on periodontal conditions. Some studies have demonstrated an improvement in periodontal status following BMS (13, 14), whereas others have shown a progress in periodontal degradation after BMS (6, 15). Furthermore, it is unclear whether there is difference in the impact of SG on the periodontal status of patients with and without T2D.

Based on a prospective cohort of patients scheduled for SG, the main aim of the current study was to determine the impact of SG on the periodontal status of patients with and without T2D in a real-world setting.

## 2 Research design and methods

### 2.1 Study design and patients

This prospective, longitudinal, two-armed cohort study was approved by the Ethics Committee on Scientific Research of Shandong University Qilu Hospital (KYLL-202111-137-1), registered at the Chinese Clinical Trial Registry (www.chictr.org.cn) with the identification number ChiCTR2200060644 (diabetic cohort) and ChiCTR2200058242 (non-diabetic cohort), and conducted in accordance with the standards set by the Declaration of Helsinki.

From April 2022 to December 2022, candidates were approached for eligibility if they were scheduled for SG at Qilu Hospital of Shandong University. SG was indicated if a patient had a body mass index (BMI) ≥ 32.5 kg/m^2^, or BMI ≥ 28 kg/m^2^ with obesity-related complications or with more than 2 metabolic syndrome components, and there were no other contraindications for general anesthesia or the surgical procedure.

The inclusion criteria of the current study were (1) body mass index (BMI) ≥ 32.5 kg/m^2^ (2); aged 18-60 years (3); with a diagnosis of periodontitis identified as a threshold of interproximal clinical attachment loss (CAL) of ≥2 mm, or ≥3 mm at ≥2 non‐adjacent teeth (16) (4); at least 16 remaining teeth (5); agree to participate in this study and sign the informed consent form.

The key exclusion criteria included individuals (1) with active smoking within the past 6 months (2); were pregnant or still in lactation (3); had a history of radiotherapy to the head and neck (4); with long-term use of nonsteroidal anti-inflammatory drugs or corticosteroids (5); with any periodontal therapy less than 6 months before the study (6); had acute infection of the affected tooth (7); had poor anesthesia or surgery tolerance because of severe cardiopulmonary dysfunction; and (8) who were unwilling to comply with follow-up visits.

Among patients included, individuals with T2D were assigned to the DM group if they had a fasting plasma glucose (FPG) level ≥7.0 mmol/l or a glycated hemoglobin (HbA1c) level ≥6.5% (10). Participants without diabetes were included and assigned to the Control group if their FPG was <6.1 mmol/L and their HbA1c level was <6.0% without taking any anti-diabetes medications or insulin.

### 2.2 Baseline measurements and biochemical analysis

Clinical determinations, including demographic features, anthropometric measurements, and laboratory test results, were obtained at baseline before surgery. Blood lipid profiles and plasma glucose levels were measured using a Roche Cobas 8000 modular analyzer system (Roche Diagnostics, IN, USA). Plasma insulin levels were determined by a two-site enzymometric assay using a Tosoh 2000 auto-analyzer (Tosoh Corp., Tokyo, Japan). The levels of inflammatory markers, including high sensitivity C-reactive protein (hs-CRP), interleukin-6 (IL-6), and tumor necrosis factor alpha (TNF-α), were determined with high sensitivity enzyme linked immunosorbent assay kits (R&D Systems). The homeostatic model assessment of insulin resistance (HOMA-IR) was calculated as fasting insulin (mU/mL) × FPG (mmol/L)/22.5 (17).

### 2.3 Oral clinical evaluation

The oral clinical examination was performed by an experienced periodontist. The following periodontal parameters were recorded for all the teeth during each examination: probing depth (PD), bleeding index (BI), plaque index (PLI), and CAL. PD was determined by measuring the distance from the gingival margin to the bottom of the gingival crevice in millimeters (14). CAL was measured as the distance from the cement–enamel junction to the bottom of the gingival crevice in millimeters (14). PLI used the standard proposed by Silness and Loe (18). The scoring method of BI was in accordance with the standard proposed by Mazza in 1981 (19).

PD and CAL were measured at six sites (mesiobuccal, midbuccal, distobuccal, distolingual, midlingual and mesiolingual) for all present teeth (except for third molars) to the nearest millimeter using a graded probe (Hu-Friedy, Chicago, IL). BI and PLI were measured at two sites (midbuccal and midlingual) per tooth.

### 2.4 Procedure

All SG operations were performed by the same experienced surgical team as described previously (10). In brief, a tubular sleeve of stomach was created by dissecting the greater curvature starting 2–4 cm from the pylorus and extending up to the angle of His.

### 2.5 Follow-up and outcomes

Suggestions for diet and physical activities but not oral hygiene were provided at the time of discharge. A reassessment of periodontal status was performed by the same examiner at 6 and 12 months after SG. The primary outcome of the current study was the periodontal condition of patients at 12 months after SG. The secondary outcomes included weight loss, diabetes remission, and alterations in inflammatory markers for up to 1 year after SG. The weight loss effect was evaluated by both percentage of total weight loss (TWL%) and excess weight loss (EWL%), which was calculated as reported previously (20). Diabetes remission was defined as HbA1c < 6.5% and the absence of antidiabetes medications for at least 2 months (21).

### 2.6 Statistical analysis

SPSS version 26.0 (SPSS Inc., Chicago, IL, USA) was used for the statistical analysis. Continuous variables are presented as the mean ± standard deviation (SD). The unpaired t tests or Mann‒Whitney tests was used to compare the mean values between baseline and after surgery and also between the two groups. The levels of TWL% and EWL% over time after surgery were analyzed using two-way (repeated-measures) ANOVA, followed by the Bonferroni *post hoc* test, and the results are reported as *^A^P* by group, *^B^P* over time, and *^C^P* due to the interaction of the two factors. The data of a small number of patients who were lost to follow-up were omitted from subsequent analysis. A *P* value less than 0.05 indicated a statistically significant difference.

## 3 Results

### 3.1 Patient characteristics

**Figure 1:**
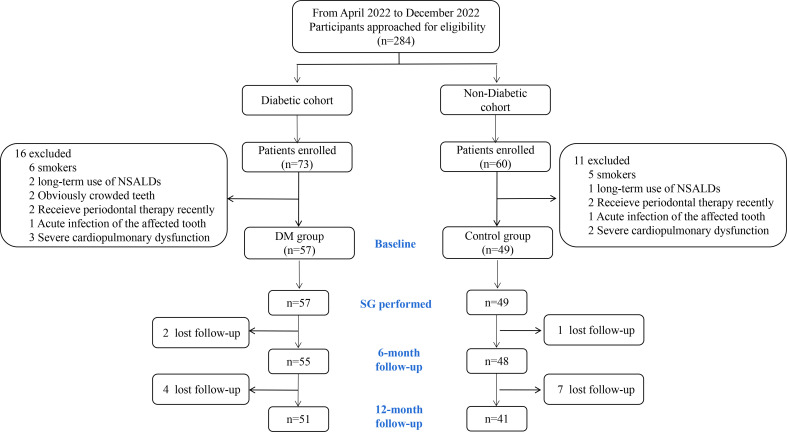
Flowchart of participants. NSALD, non-steroidal anti-inflammatory drug; SG, sleeve gastrectomy.

From April 2022 to December 2022, 284 patients scheduled for SG were approached for eligibility. 73 and 60 patients were enrolled (as shown in **Figure 1**) from the diabetic and non-diabetic cohort, respectively. After exclusion, the remaining 57 and 49 patients were included in the DM and the Control group, and 51 (92.73%) and 41 (83.67%) of which completed the 12-month follow-up, respectively (**Figure 1**). The baseline characteristics of the patients included are detailed in **Table 1**. No significant difference was detected between the DM and the Control groups in age, sex, BMI, obesity comorbidities, or tooth brushing habits. In the DM group, 42.11% of the patients were already on treatment for T2D, and only 4 patients were receiving insulin therapy.

**Table 1:** Baseline characteristics of patients.

Parameters	DM (n = 57)	Control (n = 49)	*P* value
Age (years)	32.16 ± 6.50	31.73 ± 6.44	0.733
Sex			0.728
Female	33 (57.89)	30 (61.22)	
Male	24 (42.11)	19 (38.78)	
BMI (kg/m^2^)	42.48 ± 7.33	41.87 ± 7.63	0.676
BMI-based classes, n (%)			0.900
32.5 ≤ BMI < 37.5	17 (29.82)	15 (30.61)	
37.5 ≤ BMI < 50	30 (52.63)	27 (55.10)	
BMI ≥ 50	10 (17.54)	7 (14.29)	
Obesity comorbidities			
Hypertension, n (%)	22 (38.60)	17 (34.69)	0.678
Obstructive sleep, n (%) apnea-hypopnea syndrome, n (%)	34 (59.65)	26 (53.06)	0.495
Tooth brushing			0.859
≤1 time/day	16 (28.07)	13 (26.53)	
≥2 times/day	41 (71.93)	36 (73.47)	
Fasting plasma glucose (mmol/L)	8.49 ± 3.39	5.04 ± 0.54	< 0.001
HbA1c (%)	7.71 ± 2.24	5.37 ± 0.28	< 0.001
Diabetes treatment, n (%)	24 (42.11)	–	–
On insulin therapy, n (%)	4 (7.02)	–	–

Data are presented as mean ± SD or n (%). P value refers to the statistical difference DM vs. Control.

BMI, body mass index; HbA1c, glycated hemoglobin.

### 3.2 Effects of SG on weight loss

**Figure 2:**
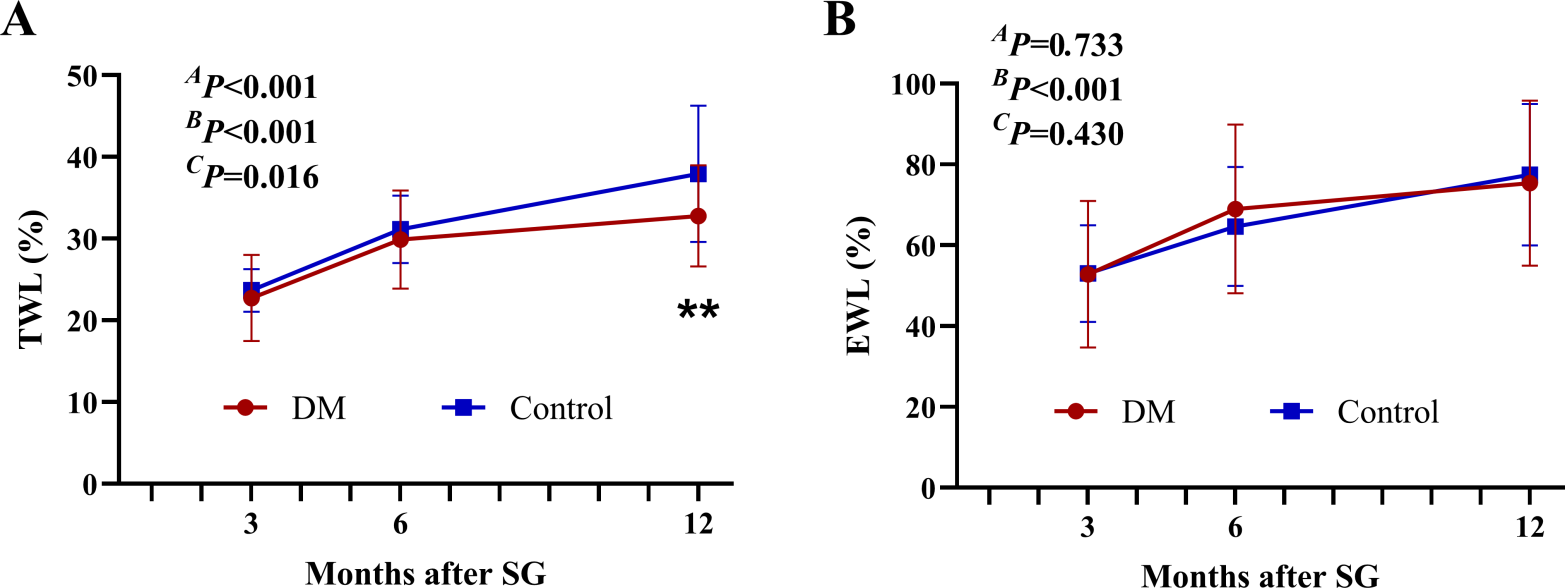
Weight-loss effect after SG. The TWL% **(A)** and EWL% **(B)** after SG. ^**^ *P* < 0.01 DM vs. Control. *^A^P* by group, *^B^P* over time, and *^C^P* due to the interaction of the two factors in the two-way ANOVA. TWL%, percentage of total weight loss; EWL%, percentage of excess weight loss; SG, sleeve gastrectomy.

The TWL% and EWL% continued to increase for up to 1 year after SG in both groups (**Figure 2**). Although both groups had comparable EWL% after surgery (*^A^P* = 0.733), patients in the DM group achieved less TWL% at 12 months after SG (32.79 ± 6.20% vs. 37.95 ± 8.34, *P* < 0.01).

### 3.3 Effects of SG on glucose homeostasis

At baseline, patients in the DM group demonstrated higher levels of HbA1c, FPG, and HOMA-IR than those in the Control group (**Table 2**). After surgery, both groups showed a significant decrease in HbA1c, FPG, fasting insulin, and HOMA-IR. At 12 months after surgery, 48 of the 51 patients (94.11%) achieved remission of diabetes, and the remaining 3 patients still needed oral medication. No significant difference was detected between groups at 12 months after SG except lower level of FPG in the Control group (4.06 ± 0.56 vs. 4.49 ± 0.59, *P* < 0.01).

**Table 2:** Glucose homeostasis before and after SG.

Parameters	Baseline		6 months after SG			12 months after SG		
	n	Mean ± SD	n	Mean ± SD	*^a^P* value	n	Mean ± SD	*^b^P* value
HbA1c (%)								
DM	57	7.71 ± 2.24**	55	5.02 ± 0.82	< 0.001	51	5.06 ± 0.87	< 0.001
Control	49	5.37 ± 0.28	48	5.11 ± 0.21	< 0.001	41	5.03 ± 0.24	< 0.001
FPG (mmol/L)								
DM	57	8.49 ± 3.39**	55	4.66 ± 0.67**	< 0.001	51	4.49 ± 0.59**	< 0.001
Control	49	5.04 ± 0.54	48	4.28 ± 0.70	< 0.001	41	4.06 ± 0.56	< 0.001
Fasting insulin (mIU/L)								
DM	57	32.54 ± 24.53	55	10.05 ± 7.53	< 0.001	51	7.54 ± 4.90	< 0.001
Control	49	26.52 ± 12.52	48	8.97 ± 5.42	< 0.001	41	6.77 ± 4.02	< 0.001
HOMA-IR								
DM	57	12.23 ± 10.31**	55	1.97 ± 1.12	< 0.001	51	1.46 ± 0.75	< 0.001
Control	49	5.91 ± 3.98	48	1.71 ± 0.79	< 0.001	41	1.22 ± 0.41	< 0.001

Data are presented as mean ± SD. ^a^P value refers to the statistical difference 6 months after SG vs. baseline. ^b^P value refers to the statistical difference 12 months after SG vs. baseline. **P < 0.01 DM vs. control.

HbA1c, glycated hemoglobin; FPG, fasting plasma glucose; HOMA-IR, the homeostatic model assessment of insulin resistance.

### 3.4 Effects of SG on blood lipid profiles

Before surgery, patients in the DM group showed significantly higher level of triglycerides than those in the Control group (2.42 ± 1.34 vs. 1.49 ± 0.60, *P* < 0.01, **Table 3**). Patients in both the DM group and the Control group showed an increased level of high-density lipoprotein-cholesterol (HDL-c) as well as decreased levels of triglycerides, low-density lipoprotein-cholesterol (LDL-c), and nonesterified fatty acids (NEFA) after SG. No significant difference was detected in level of total cholesterol between baseline and 6 or 12 months after SG in neither group. At 12 months after SG, patients in the Control group achieved better improvement in levels of total cholesterol (4.25 ± 1.17 vs. 4.84 ± 0.96, *P* < 0.05) and LDL-c (2.35 ± 0.77 vs. 2.76 ± 0.38, *P* < 0.01) compared with those in the DM group.

**Table 3:** Lipid profiles before and after SG.

Parameters	Baseline		6 months after SG			12 months after SG		
	n	Mean ± SD	n	Mean ± SD	*^a^P* value	n	Mean ± SD	*^b^P* value
Triglycerides (mmol/L)								
DM	57	2.42 ± 1.34**	55	1.17 ± 0.45	< 0.001	51	0.89 ± 0.31	< 0.001
Control	49	1.49 ± 0.60	48	1.01 ± 0.42	< 0.001	41	0.87 ± 0.45	< 0.001
Total cholesterol (mmol/L)								
DM	57	5.19 ± 1.45	55	4.96 ± 1.22	0.367	51	4.84 ± 0.96*	0.147
Control	49	4.76 ± 1.28	48	4.92 ± 1.21	0.528	41	4.25 ± 1.17	0.052
LDL-c (mmol/L)								
DM	57	3.03 ± 0.85	55	3.11 ± 1.03	0.654	51	2.76 ± 0.38**	0.039
Control	49	2.97 ± 0.89	48	2.93 ± 0.86	0.822	41	2.35 ± 0.77	< 0.001
HDL-c (mmol/L)								
DM	57	1.13 ± 0.49	55	1.30 ± 0.34	0.036	51	1.43 ± 0.22	< 0.001
Control	49	1.15 ± 0.47	48	1.31 ± 0.54	0.123	41	1.41 ± 0.38	0.005
NEFA (umol/dL)								
DM	57	83.92 ± 28.02	55	75.06 ± 20.02	0.058	51	58.73 ± 18.45	< 0.001
Control	49	85.65 ± 37.06	48	77.46 ± 32.11	0.247	41	62.44 ± 31.46	0.002

Data are presented as mean ± SD. ^a^P value refers to the statistical difference 6 months after SG vs. baseline. ^b^P value refers to the statistical difference 12 months after SG vs. baseline. *P < 0.05, **P < 0.01 DM vs. control.

LDL-c, low-density lipoprotein-cholesterol; HDL-c, high-density lipoprotein-cholesterol; NEFA, nonesterified fatty acids.

### 3.5 Impact of SG on the periodontal status of patients

At baseline, patients in the DM group had higher levels of PD (4.10 ± 1.08 vs. 3.68 ± 0.87, *P* < 0.05), BI (2.67 ± 0.68 vs. 2.13 ± 0.71, *P* < 0.01), and CAL (2.04 ± 0.96 vs. 1.69 ± 0.61, *P* < 0.05) than those in the Control group (**Table 4**), indicating the worse periodontal condition in patients with T2D than those without. No significant difference was detected in the level of PLI between the two groups before surgery. Compared with those at baseline, significantly lower PLI and BI values were detected after SG in both the DM and the Control group. No statistically significant change was observed in PD or CAL after SG neither in the DM group nor in the Control group. At 12 months after SG, the DM group had similar levels of PLI, BI, and CAL but higher level of PD (4.08 ± 0.92 vs. 3.63 ± 1.03, *P* < 0.05) compared with the Control group.

**Table 4:** Comparison of periodontal status before and after SG.

Parameters	Baseline		6 months after SG			12 months after SG		
	n	Mean ± SD	n	Mean ± SD	*^a^P* value	n	Mean ± SD	*^b^P* value
PLI								
DM	57	2.20 ± 0.43	55	1.74 ± 0.37	< 0.001	51	1.78 ± 0.35	< 0.001
Control	49	2.05 ± 0.37	48	1.79 ± 0.44	0.002	41	1.85 ± 0.32	0.008
PD (mm)								
DM	57	4.10 ± 1.08*	55	4.12 ± 0.96	0.918	51	4.08 ± 0.92*	0.918
Control	49	3.68 ± 0.87	48	3.77 ± 0.92	0.622	41	3.63 ± 1.03	0.803
BI								
DM	57	2.67 ± 0.68**	55	2.09 ± 0.49	< 0.001	51	2.05 ± 0.48	< 0.001
Control	49	2.13 ± 0.71	48	1.82 ± 0.52	0.016	41	1.88 ± 0.39	0.047
CAL (mm)								
DM	57	2.04 ± 0.96*	55	2.05 ± 1.12	0.960	51	2.02 ± 1.14	0.921
Control	49	1.69 ± 0.61	48	1.75 ± 0.73	0.661	41	1.66 ± 0.57	0.811

Data are presented as mean ± SD. ^a^P value refers to the statistical difference 6 months after SG vs. baseline. ^b^P value refers to the statistical difference 12 months after SG vs. baseline. *P < 0.05, **P < 0.01 DM vs. control.

PLI, plaque index; PD, probing depth; BI, bleeding index; CAL, clinical attachment loss.

### 3.6 Changes in inflammatory markers after SG

As shown in **Table 5**, the DM group showed higher levels of hs-CRP and IL-6 compared with the Control group at baseline. However, significant difference was detected only in IL-6 (4.21 ± 2.11 vs. 3.36 ± 1.91, *P* < 0.05). Both groups showed significantly lower levels of hs-CRP and IL-6 as well as similar level of TNF-α after SG than at baseline. At 12 months after SG, no significant difference was detected between the two groups in levels of hs-CRP, IL-6, or TNF-α.

**Table 5:** Levels of inflammatory markers before and after SG.

Parameters	Baseline		6 months after SG			12 months after SG		
	n	Mean ± SD	n	Mean ± SD	*^a^P* value	n	Mean ± SD	*^b^P* value
hs-CRP (mg/L)								
DM	57	12.37 ± 9.14	55	5.65 ± 4.43	< 0.001	51	5.17 ± 4.51	< 0.001
Control	49	9.51 ± 7.27	48	4.93 ± 3.72	<0.001	41	5.21 ± 4.01	< 0.001
IL-6 (pg/ml)								
DM	57	4.21 ± 2.11*	55	2.79 ± 1.61	< 0.001	51	2.93 ± 1.95	0.002
Control	49	3.36 ± 1.91	48	2.65 ± 1.29	0.035	41	2.37 ± 1.34	< 0.001
TNF-α (pg/ml)								
DM	57	11.01 ± 7.43	55	10.54 ± 8.24	0.752	51	11.36 ± 6.41	0.795
Control	49	11.73 ± 6.86	48	11.46 ± 7.16	0.850	41	11.23 ± 5.25	0.697

Data are presented as mean ± SD. ^a^P value refers to the statistical difference 6 months after SG vs. baseline. ^b^P value refers to the statistical difference 12 months after SG vs. baseline. *P < 0.05 DM vs. control.

hs-CRP, high sensitive C-reactive protein; IL-6, interleukin-6; TNF-α, tumour necrosis factor alpha.

## 4 Discussion

Obesity and T2D are two of the most common chronic diseases worldwide. Cross-sectional studies supported that there was a strong connection between periodontitis and obesity or T2D (9). As the most commonly performed BMS procedure, SG has been confirmed to be more effective than conventional medical therapy for long-term weight loss and control of T2D (22, 23). However, further studies are needed to elucidate the impact of SG on the periodontal status of patients with and without T2D.

The principal findings of the present study were as follows: 1. For patients with obesity and scheduled for SG, those with T2D had further worsened periodontal status compared with those without; 2. Both patients with and without T2D demonstrated improved periodontal status for up to 1 year after SG, accompanied by weight loss and glucose reduction; 3. Even patients with T2D achieved less TWL% after surgery than those without, their periodontal condition had more substantial improvement with no significant difference in PLI and BI between groups at 1 year after SG; 4. The significant reduction in the inflammatory biomarkers, including hs-CRP and IL-6, contributed to the improvement of periodontal status after SG in both groups.

The patients in the present study consisted of mainly females, with a mean age of approximately 32 years. Previous studies have confirmed that tobacco smoking has a detrimental effect on the incidence and progression of periodontitis (24), so participants with active smoking within the past 6 months were excluded from the current study. 28.07% of patients with T2D and 24.40% of patients without T2D had a tooth brushing ≤1 time/day, indicating a low level of attention to oral hygiene in the patients included. In addition, only 42.11% of patients with T2D were already on treatment, suggesting the poor awareness towards personal health among the participants.

Our study further confirmed the weight loss effect of SG with more than 30% TWL at 1 year after surgery in both groups. A previous meta-analysis including 6235 Asian patients reported 32.1% TWL at 1 year after SG (25). Our previous research demonstrated that, male and female patients with balanced baseline BMI could achieve comparable TWL% for up to 1 year after SG (20). In addition, out results showed that patients with T2D achieved less TWL% after surgery than those without, which is similar to our previous report (10). Another study performed by Diedisheim et al. demonstrated subjects with obesity and T2D who have poor pre-operative glycemic control displayed reduced weight-loss and less improvement in body composition compared to patients with obesity but without T2D (26). These results suggest that even though the rapid and sustained weight loss effect of BMS has been confirmed, glycemic control prior to surgery is an important factor to be taken into account in the expectation of the weight loss outcome after surgery.

Previous studies have confirmed that T2D is associated with a higher prevalence, incidence, severity, and progression of periodontitis (9, 27, 28). Patients with poorly controlled diabetes have more serious clinical symptoms of periodontitis compared with those without diabetes or well controlled diabetes (29, 30). A study from Italian participants demonstrated that the probability of severe periodontitis in patients increased by approximately 60% for 1 unit of increase in serum HbAlc (30). However, it should be noted that the evidence abovementioned focused on the general population, with some overweight individuals involved at most. There has been no evidence yet for patients with pathological obesity, especially those who meet the indications for BMS. The present study filled in the blank of this area that we demonstrated more serious clinical symptoms of periodontitis (with higher PD, BI, and CAL) in patients scheduled for SG with T2D compared with those without diabetes. PLI is a parameter that closely related to the individual oral hygiene habits (31). There was no significant difference in tooth brushing between the DM group and the Control group, and that could be the reason why there was similar levels of PLI between groups at baseline. The plausible pathogenic mechanisms of T2D promoting the susceptibility of periodontitis included microbiome factors, enhanced inflammatory response via cytokines or adipokines, host immune factors, and oxidative stress (32).

In addition to the improvements in systemic conditions (glucose homeostasis, insulin sensitivity, and plasma lipid profiles), the present study revealed a positive impact of SG on periodontal status in both the DM and the Control group characterized by a decrease in periodontal inflammation and an improvement of oral hygiene situation, which was reflected by the decrease in BI and PLI, respectively. Jaiswal et al. reported improvements in the bleeding score and PLI 6 months after BMS and attributed to changes in diet and oral hygiene instructions provided to patients before surgery (13). However, another prospective study conducted by Sales-Peres et al. demonstrated significantly increased gingival bleeding and unaltered CAL 6 months after BMS, suggesting the deterioration of periodontal conditions after bariatric surgery (33). The discrepancy among studies may be attributed to differences in individual characteristics and oral hygiene intervention. As far as we know, there has been no existing evidence comparing the periodontal status between patients with and without diabetes after BMS. Our results confirmed that both patients with and without T2D could benefit from SG in terms of periodontal status in the absence of any instructions for oral hygiene. In addition, our results showed no obvious change in PD or CAL after SG. The reason could be that these parameters often change only after periodontal surgical treatment (13), which was not involved in this study.

The real mechanisms underlying the improvement in periodontal status after SG in patients with T2D are not well understood. Both obesity and T2D are characterized by persistent, low-grade inflammation (34). Low–grade systemic inflammation has also been proposed as a potential causative factor for periodontitis (35). The most important mediators related to this association are TNF–α and IL–6, which may alter the activities of leukocytes, osteoblasts and osteoclasts, causing periodontal tissue destruction (36). The present study demonstrated significant higher level of IL-6 in the DM group before surgery. The result is consistent with a previous report by Rodrigues et al., in which they found higher levels of IL-6 and hs-CRP in patients with T2D, especially in those with BMI > 30 kg/m^2^ (37). In addition, our study demonstrated a significantly decrease in levels of hs-CRP and IL-6 after SG than at baseline in both groups. Mallipedhi et al. investigated changes in inflammatory markers after SG in patients with impaired glucose homeostasis and T2D, and reached the same conclusion as ours (38). In addition, they also observed a significant improvement in leptin as well as unaltered adiponectin and IL-10 after SG.

The major strengths of the current study are that we compared the impact of SG on the periodontal status of patients with and without T2D based on a prospective cohort in a real-world setting. However, this study has several limitations. First, information on individual oral habits after surgery was not involved. However, with no additional instructions for oral hygiene was provided at the time of discharge or in the process of follow-up, the possible individual behavioral changes should originate from the surgery or its related other improvements. Second, the generalizability of the results is limited by the single-center design and the relatively short 1-year follow-up period.

## 5 Conclusion

In conclusion, both patients with and without T2D demonstrated improved periodontal status for up to 1 year after SG. Patients with T2D achieved less weight loss but a more substantial improvement in periodontal condition. The significant reduction in inflammatory biomarkers contributed to the improvement of periodontal status after SG.

## Data Availability

The raw data supporting the conclusions of this article will be made available by the authors, without undue reservation.
